# GFAp and tau protein as predictors of neurological outcome after out-of-hospital cardiac arrest: A post hoc analysis of the COMACARE trial

**DOI:** 10.1016/j.resuscitation.2021.11.033

**Published:** 2022-01

**Authors:** Jaana Humaloja, Marika Lähde, Nicholas J Ashton, Reinikainen Matti, Johanna Hästbacka, Pekka Jakkula, Hans Friberg, Tobias Cronberg, Ville Pettilä, Kaj Blennow, Henrik Zetterberg, Markus B Skrifvars, COMACARE Study Groups

**Affiliations:** a Department of Emergency Care and Services, University of Helsinki and Helsinki University Hospital, Helsinki, Finland; b Department of Anesthesiology and Intensive Care, Päijät-Häme Central Hospital, Lahti, Finland; c Department of Psychiatry and Neurochemistry, Institute of Neuroscience and Physiology, the Sahlgrenska Academy at the University of Gothenburg, Mölndal, Sweden; d Department of Anesthesiology and Intensive Care, University of Eastern Finland and Kuopio University Hospital, Kuopio, Finland; e Department of Anesthesiology, Intensive Care, and Pain Medicine, Helsinki University and Helsinki University Hospital, Helsinki, Finland; f Department of Clinical Sciences, Lund, Anaesthesia and Intensive Care, Lund University, Skåne University Hospital, Malmö, Sweden; g Department of Clinical Sciences Lund, Neurology, Lund University, Skåne University Hospital, Lund, Sweden; h Clinical Neurochemistry Laboratory, Sahlgrenska University Hospital, Mölndal, Sweden; i Department of Neurodegenerative Disease, UCL Institute of Neurology, Queen Square, London, UK; j UK Dementia Research Institute at UCL, London, UK; k Hong Kong Center for Neurodegenerative Diseases, Hong Kong, China

**Keywords:** Out-of-hospital cardiac arrest, Biomarkers, Tau protein, Glial fibrillary acidic protein, Neurological outcome prognostication

## Abstract

**Aim:**

To determine the ability of serum glial fibrillary acidic protein (GFAp) and tau protein to predict neurological outcome after out-of-hospital cardiac arrest (OHCA).

**Methods:**

We measured plasma concentrations of GFAp and tau of patients included in the previously published COMACARE trial (NCT02698917) on intensive care unit admission and at 24, 48, and 72 h after OHCA, and compared them to neuron specific enolase (NSE). NSE concentrations were determined already during the original trial. We defined unfavourable outcome as a cerebral performance category (CPC) score of 3–5 six months after OHCA. We determined the prognostic accuracy of GFAp and tau using the receiver operating characteristic curve and area under the curve (AUROC).

**Results:**

Overall, 39/112 (35%) patients had unfavourable outcomes. Over time, both markers were evidently higher in the unfavourable outcome group (p < 0.001). At 48 h, the median (interquartile range) GFAp concentration was 1514 (886–4995) in the unfavourable versus 238 (135–463) pg/ml in the favourable outcome group (p < 0.001). The corresponding tau concentrations were 99.6 (14.5–352) and 3.0 (2.2–4.8) pg/ml (p < 0.001). AUROCs at 48 and 72 h were 0.91 (95% confidence interval 0.85–0.97) and 0.91 (0.85–0.96) for GFAp and 0.93 (0.86–0.99) and 0.95 (0.89–1.00) for tau. Corresponding AUROCs for NSE were 0.86 (0.79–0.94) and 0.90 (0.82–0.97). The difference between the prognostic accuracies of GFAp or tau and NSE were not statistically significant.

**Conclusions:**

At 48 and 72 h, serum both GFAp and tau demonstrated excellent accuracy in predicting outcomes after OHCA but were not superior to NSE.

**Clinical trial registration:**

NCT02698917 (https://www.clinicaltrials.gov/ct2/show/NCT02698917).

## Introduction

Hypoxic-ischemic brain injury is considered the major determinant of neurological outcome after cardiac arrest (CA).1., 2. The expected outcome should guide intensive care treatment efforts to those who are likely to benefit. However, the long-term outcome is difficult to predict during the early days of post-resuscitation. Resuscitation guidelines suggest a multimodal approach for outcome prediction utilising clinical examination, brain imaging, electroencephalography, and laboratory biomarkers.3., 4. Currently neuron specific enolase (NSE) is the only biomarker recommended by the guidelines. In a recent meta-analysis, NSE showed good accuracy in predicting the outcome beyond 24 h after CA with pooled area under the receiver operating characteristic curve (AUROC) of 0.92.^5^ However, NSE has confounding sources, it is prone to sample haemolysis, and the cutoff values for poor prognosis vary widely among studies.^5^ Therefore, more accurate blood biomarkers for neurological prognostication after CA are needed. The serum level of glial fibrillary acidic protein (GFAp) has been recognised as predictor of neurological outcome after head trauma; elevated concentrations have also been measured after stroke, subarachnoid haemorrhage, and CA.6., 7., 8., 9., 10., 11., 12. GFAp is an intermediate-filament component of the astrocytic cytoskeleton highly specific to the central nervous system.^13^ Meanwhile, the tau protein forms microtubule-stabilising structures and is primarily found in axons in central nervous tissues.14., 15. Elevated tau concentrations in blood have been reported after ischemic stroke and CA. Moreover, tau concentrations increase in the cerebrospinal fluid of patients with traumatic brain injury or neurodegenerative diseases, particularly Creutzfeldt – Jakob disease and Alzheimer’s disease.16., 17., 18., 19., 20., 21.

In this study, we determined the accuracy of GFAp and tau in prediction of neurological outcome after out-of-hospital cardiac arrest (OHCA) utilising blood samples collected in the previously published COMACARE trial (NCT02698917).^22^ We hypothesised that both GFAp and tau would be associated with the neurological outcomes and they would function as potential prognostic tools after CA. In addition, given the difference of their origin in brain tissues, we hypothesised that the combination of both biomarkers would further improve the predictive accuracy.

## Methods

### Trial design and study population

This study utilised blood samples collected in the COMACARE trial from March 2016 to November 2017. The participants in this sub-study of the trial were from six different intensive care units (ICU) in Finland. The trial protocol and main findings have been published previously.22., 23. In brief, the trial was a prospective study of 120 OHCA patients randomised with a 2^3^ factorial design to normal or moderately elevated arterial oxygen tension (PaO_2_ 10–15 or 20–25 kPa), low-normal or high-normal arterial carbon dioxide tension (PaCO_2_ 4.5–4.7 or 5.8–6.0 kPa), and low-normal or high-normal mean arterial pressure (65–75 or 80–100 mmHg) for the first 36 h of ICU treatment. The participants included adult patients (aged 18–80) resuscitated from witnessed OHCA, with initial shockable rhythm, and who were comatose and mechanically ventilated on ICU admission. All patients received targeted temperature management (TTM) at 33 or 36 °C. European Resuscitation Council and European Society of Intensive Care Medicine guidelines were followed in the neurological prognostication.^24^ The aim of the COMACARE trial was to determine the effects of post-arrest treatment targets on neurological damage markers, primarily NSE, at 48 h after CA. The current study determined the concentrations of GFAp and tau in peripheral blood. The Northern Savo Hospital District research ethics committee approved the original trial protocol (Decision No. 295/13.02.00/2015, 23 February 2016) and an amendment for the current analysis (7 June 2019). The trial was performed in accordance with the Declaration of Helsinki.

### GFAp and tau concentrations and the outcome measure

Blood samples for GFAp and tau determination were collected on ICU admission (0 h) and at 24, 48, and 72 h thereafter. After centrifugation the plasma samples were immediately frozen to −70 °C. The samples were analysed in September 2019 using commercially available single-molecule array (Simoa) immunoassays (Quanterix, Billerica, MA) at the Clinical Neurochemistry Laboratory of Sahlgrenska University Hospital (Mölndal, Sweden).^25^ The measurements were performed on an HD-1 Analyzer (Quanterix, Billerica, MA) in one round of analysis using one batch of reagents with samples from the same patients side by side on the plates. Intra-assay coefficients of variation were < 10%. The laboratory technicians were blinded to the clinical data.

The primary outcome measure was neurological outcome six months after CA determined by the Cerebral Performance Category (CPC) scale, as evaluated by a neurologist blinded to study group allocation, treatment, and laboratory results.^26^ Favourable outcome was defined as a CPC score of 1 or 2 corresponding to independence in daily activities as the minimum, whereas unfavourable outcome was defined as a score of 3 to 5 corresponding to severe cerebral disability or death.

### Statistical methods

We present continuous data as medians and interquartile ranges (IQR) and categorical data as counts and percentages. We tested all continuous variables for normality and used the Mann–Whitney U test to compare the non-normally distributed data.^27^ We compared categorical data with Pearson’s chi-squared test or Fisher’s exact test.28., 29. Through linear mixed-model analysis with compound symmetry, we compared the GFAp and tau concentrations over time between the patients with favourable vs unfavourable neurological outcomes and between patients in different treatment arms.^30^ We used several methods to evaluate the markers’ prognostic abilities. First, we assessed the ability of single-timepoint measurements of GFAp and tau to predict the six-month neurological outcome by determining the AUROC with 95% confidence interval (95% CI).^31^ NSE concentrations were determined at corresponding timepoints during the original trial, and we compared the AUROC values of GFAp, tau, and NSE with bootstrap method.^22^ Next, we created multivariable logistic regression models that included age, bystander-initiated life support, delay to return of spontaneous circulation, and NSE to predict poor neurological outcome at six months.^32^ We incorporated GFAp and tau concentrations at 0, 24, 48, and 72 h alternately into the model and determined the prognostic accuracy improvements by comparing the AUROCs of the baseline model and the models with added markers. We determined the cutoff values of GFAp and tau for poor prognosis through the Youden method and with high specificities (95%, 97%, and 99%, corresponding to false positive rates of 5%, 3%, and 1% respectively) based on the receiver operating characteristic curve (ROC) coordinate points.33., 34. Moreover, we determined the positive predictive value, negative predictive value, and positive likelihood ratios for these cutoff values. Additionally, to identify a threshold below which most or all of the patients had favourable outcomes, we determined cutoffs with sensitivities of 95% and 100% for unfavourable outcome (corresponding to a false negative rate of 5% and 0%, respectively). Lastly, based on the measured concentrations of GFAp and tau at 48 and 72 h, we determined how the corresponding cutoff values predicting poor outcome with 95% specificity categorised the patients with favourable vs unfavourable outcomes in three scenarios: a) both GFAp and tau concentrations are below the cutoff (both suggesting favourable outcomes), b) either GFAp or tau concentration is above the cutoff (“grey area”), and c) both GFAp and tau concentrations are above the cutoff (both suggesting unfavourable outcomes). We conducted all statistical analyses with IBM SPSS version 27.0.1.0, RStudio version 1.4.1717, and GraphPad Prism version 9.1.2 for MacOs.

## Results

**Fig. 1:**
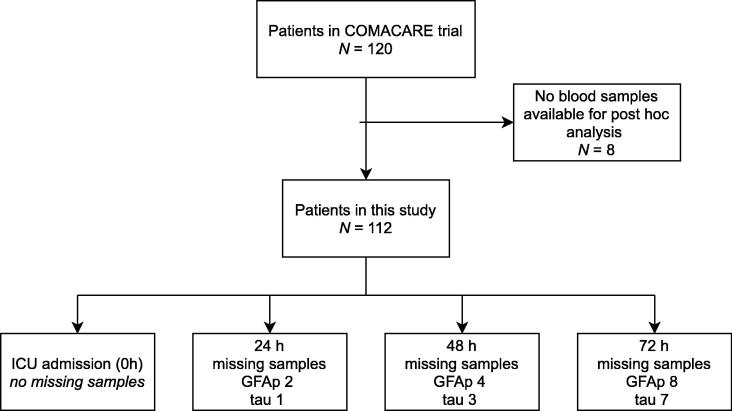
Flowchart of the study population and blood samples available for the analyses. Definitions of abbreviations: COMACARE trial: Carbon dioxide, Oxygen, and Mean Arterial pressure After Cardiac Arrest and Resuscitation trial ICU: intensive care unit GFAp: glial fibrillary acidic protein.

Blood samples were available for 112 patients. The flowchart of patient inclusion in every sub-analysis is illustrated in Fig. 1. The median (IQR) patient age was 62 (53–68) and 92 (82%) of the participants were male. Table 1 presents the baseline characteristics of the patients included in the study and those who were excluded.

**Table 1:** Baseline characteristics of the study population.

	Patients included N = 112	Patients excluded N = 8	P*	Data missing *N*
	Median (IQR)/n (%)			
Age	62 (53–68)	51 (46–71)	0.32	
Male sex	92 (82)	6 (75)	0.64	
BMI	26.3 (23.8–29.4)	26.2 (21.5–28.2)	0.70	2
Current smoker	35 (31)	5 (63)	0.15	13
NYHA class III or IV (before the arrest)	9 (8)	1 (13)	0.87	
Hypertension	56 (50)	4 (50)	0.64	
Bystander resuscitation **	93 (83)	5 (63)	0.16	
Delay to advanced life support (*minutes*)***	10 (7–12)	10 (7–13)	0.84	2
ROSC time (*minutes*)	21 (16–26)	15 (11–20)	0.04	
GCS on admission	3 (3–3)	3 (3–3)	0.76	9
Apache II score	28 (24–31)	26 (12–33)	0.47	
TTM target			0.06	
33 °C	75 (67)	8 (100)		
36 °C	37 (33)	0 (0)		

IQR: interquartile range.

BMI: body mass index.

NYHA: New York Heart Association.

ROSC: return of spontaneous circulation.

GCS: Glasgow Coma Scale.

APACHE: Acute Physiology and Chronic Health Evaluation.

TTM: targeted temperature management.

* Mann Whitney U/Fisher exact test.

** bystander-initiated chest compressions.

*** delay from the arrest to the arrival of paramedic or doctoral unit with advanced life support equipment and staff.

**Fig. 2:**
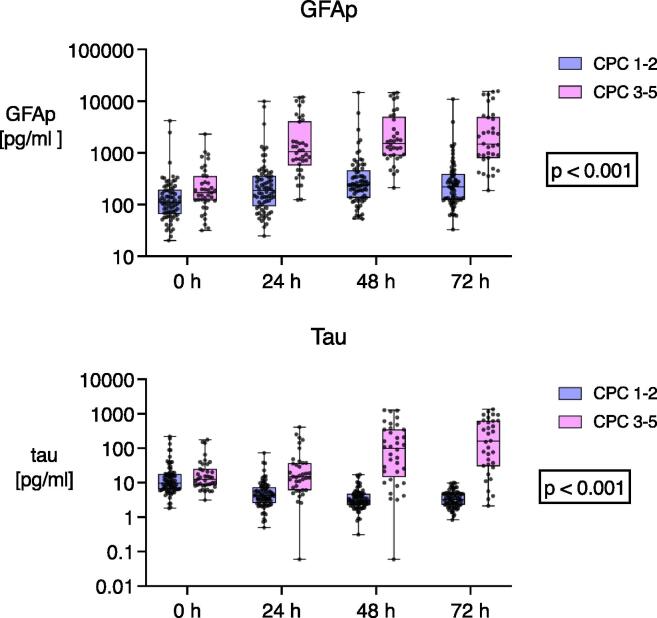
GFAp and tau concentrations between patients with favourable (CPC 1–2) and unfavourable (CPC 3–5) outcomes at six months presented on a logarithmic scale. The p-value indicates the difference of GFAp and tau concentrations over time between the outcome groups. Definitions of abbreviations: GFAp: glial fibrillary acidic protein CPC: cerebral performance category.

Overall, 39 (35%) patients had unfavourable neurological outcomes. Median GFAp and tau concentrations were significantly higher in patients with unfavourable outcomes over time and individually at all timepoints except with tau upon ICU admission (see Fig. 2, Table 2, and SM Table S1). The GFAp concentration peaked at 48 h. The median (IQR) GFAp concentration at 48 h was 1514 (886–4995) pg/ml in patients with unfavourable and 238 (135–463) pg/ml in patients with favourable outcomes (p < 0.001). In the patients with unfavourable outcomes, the median tau concentration was highest at 72 h (161 [30.2–626]) pg/ml]. In the patients with favourable outcomes, the median tau concentration was highest on admission (9.9 [6.4–18.1] pg/ml)). The GFAp and tau concentrations did not differ between the groups in the randomized treatment arms of PaO_2_, PaCO_2_, and mean arterial pressure (see SM Table S1, S2, and S3).

**Table 2:** GFAP and tau concentrations between patients with favourable (CPC 1–2) vs unfavourable (CPC 3–5) neurological outcomes at six months.

GFAp concentration pg/ml (IQR)				
Time	CPC 1–2	CPC 3–5	*p* value*	missing
	n = 73	n = 39		
0 h	110.6 (65.9–196.0)	170.9 (119.3–357.2)	0.007	0
24 h	187.7 (92.9–360.0)	1050.0 (4078.2–570.0)	<0.001	2
48 h	238.3 (134.6–463.2)	1513.7 (885.9–4994.8)	<0.001	4
72 h	219.7 (122.4–392.7)	1469.2 (788.8–4941.2)	<0.001	8

Tau concentration pg/ml (IQR)				

Time	CPC 1–2	CPC 3–5	*p* value*	missing

	n = 73	n = 39		
0h	9.9-(6.4–18.1)	12.7 (8.3–25.3)	0.14	0
24h	4.3 (2.6–7.5)	14.9 (6.1–37.3)	<0.001	1
48h	3.0 (2.2–4.8)	99.6 (14.5–352.1)	<0.001	3
72h	3.2 (2.2–4.9)	160.5 (30.2–625.7)	<0.001	7

GFAp glial fibrillary acidic protein.

CPC cerebral performance category.

IQR interquartile range.

* Mann-Whitney U.

### Prognostic accuracy of GFAp and tau and clinical prognostication data

**Fig. 3:**
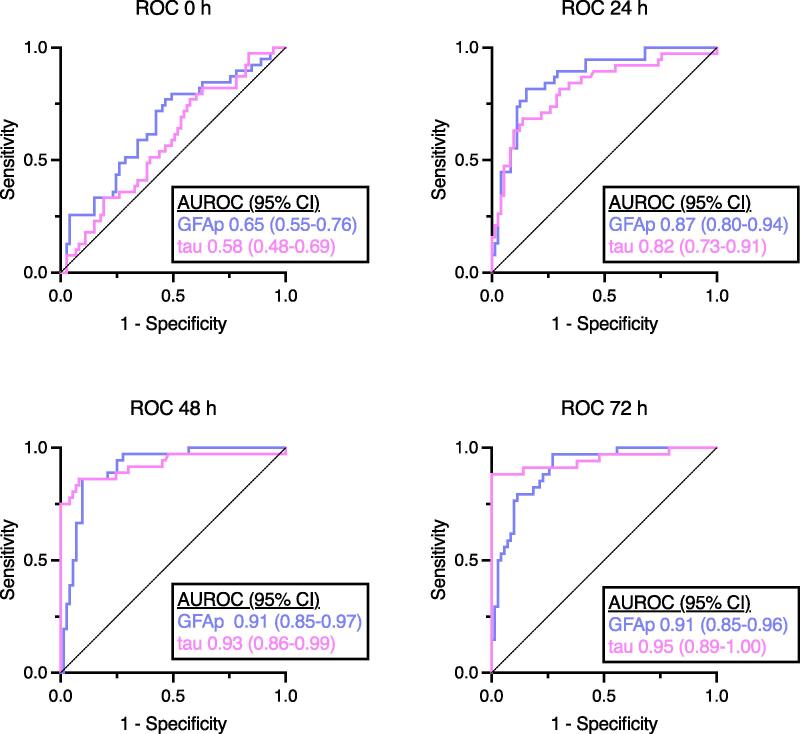
Receiver operating characteristic curves and areas under the curves (AUROC) for concentrations of GFAp and tau on ICU admission (0 h) and at 24, 48 and 72 h for prediction of favourable (CPC 1–2) vs unfavourable (CPC 3–5) outcomes six months after cardiac arrest. Definitions of abbreviations: 95% CI: 95% confidence interval ROC: receiver operating characteristic AUROC: area under the receiving operating characteristic curve GFAp: glial fibrillary acidic protein.

Prognostic accuracy of both GFAp and tau were better at later timepoints (48 and 72 h) compared to earlier timepoints (0 and 24 h). The AUROC (95% CI) for GFAp was 0.91 (0.85–0.97) at 48 and 0.91 (0.85–0.96) at 72 h, while that for tau was 0.93 (0.86–0.99) at 48 and 0.95 (0.89–1.00) at 72 h (see Fig. 3). The prognostic accuracy of tau was not superior to that of GFAp, and the prognostic accuracy of GFAp or tau was not superior to that of NSE at any time point (see SM Table S4).

The baseline logistic regression model with clinical data predicted poor outcome at six months with an AUROC of 0.87 (0.80–0.93). The AUROC values after the addition of NSE at different time points are shown in Table 3. The predictive accuracy of the model did not increase at any time point after the addition of GFAp. The addition of tau improved the model accuracy from AUROC of 0.94 (0.88–0.99) to 0.98 (0.97–1.00) at 48 h, and from 0.94 (0.89–0.99) to 0.99 (0.98–1.00) at 72 h, see Table 3. The ROC curves are presented in SM Fig. S1 and the odds ratios of the regression model in SM Table S5.

**Table 3:** Increase in the prognostic value of the logistic regression model when GFAp and tau concentrations at different time points are added to the model.

	AUROC (95 % CI)				
Time	Baseline* + NSE	Baseline* + NSE + GFAp	p**	Baseline* + NSE + tau	p***
0 h	0.86 (0.79–0.93)	0.87 (0.80–0.94)	0.41	0.87 (0.81–0.94)	0.31
24 h	0.89 (0.82–0.95)	0.91 (0.85–0.97)	0.07	0.90 (0.85–0.96)	0.18
48 h	0.94 (0.88–0.99)	0.94 (0.89–0.99)	0.51	0.98 (0.97–1.00)	0.05
72 h	0.94 (0.89–0.99)	0.94 (0.89–0.99)	0.52	0.99 (0.98–1.00)	0.03

AUROC area under the receiving operating characteristic curve.

95% CI 95% confidence interval.

NSE neuron specific enolase.

GFAp glial fibrillary acidic protein.

* Baseline model including patient age, time to return of spontaneous circulation, and bystander resuscitation.

** significance of the difference between AUROCs of baseline + NSE and baseline + NSE + GFAp.

*** significance of the difference between AUROCs of baseline + NSE and baseline + NSE + tau.

### Cutoff values for predicting good and poor outcomes

For predicting unfavourable outcome, the cutoff values at 48 h with specificities of 99% and 95% were 6262 and 1798 for GFAp, and 16.0 and 9.86 pg/ml for tau respectively. The corresponding sensitivities (95% CI) were 0.19 (0.07–0.32) and 0.39 (0.23–0.55) for GFAp, and 0.75 (0.61–0.89) and 0.81 (0.68–0.93) for tau respectively. The corresponding positive and negative predictive values and the positive likelihood ratios are shown in SM Table S6. The cutoff values predicting favourable outcome set at a sensitivity of 95% at 48 h were 439 pg/ml for GFAp and 3.28 pg/ml for tau (p < 0.001). The corresponding negative predictive values and specificities are indicated in SM Table S7.

When the measured concentrations of both GFAp and tau exceeded the determined cutoff values with 95% specificity with both markers suggesting unfavourable outcome, 12/12 (100%) at 48 h and 17/17 (100%) patients at 72 h had unfavourable six-month outcomes (see SM Fig. S2 and Table S8). When only either one of the markers were above the poor outcome cutoff, 19 (73%) patients out of 26 at 48 h and 14 (67%) patients out of 21 at 72 h truly had unfavourable six-month outcomes. The p-value for analyses in both timepoints was p < 0.001.

## Discussion

In this study, we found that both GFAp and tau are accurate predictors of neurological outcomes at six months after OHCA. The best predictive ability was observed for GFAp at 48 h and for tau at 72 h after CA. With high specificities (>95%, corresponding to false positive rates below 5%) our findings suggested poor sensitivity for GFAp, while the sensitivity for tau was very good (Table S6). The use of GFAp and tau simultaneously further improved predictive accuracy, which may indicate that they add complementary information about hypoxic-ischemic brain injury. However, in this study GFAp or tau were not superior to NSE in predicting outcome after CA (SM Table S4), which could possibly be due to our limited sample size. Thus, larger studies are warranted to fully appreciate the roles of GFAp and tau in outcome prediction after CA.

Despite the global nature of ischemia during CA, early ischemic damage locates mainly in the brain grey matter in the hippocampus, cerebellum, and brain cortex.1., 35. Ischemia-reperfusion, oedema, and inflammatory responses mediate secondary damage, which is more diffused compared with early damage.36., 37. Beyond 24 h after the arrest, severe hypoxic-ischemic encephalopathy is classically seen in computed tomography imaging as the loss of the differentiation between grey and white matter.^38^ GFAp is the major structural scaffold of the cytoskeleton in astrocytes and, hence, is more concentrated in brain grey matter.13., 39. GFAp production is upregulated following ischemia and neurotrauma, which is believed to act as defensive mechanism to handle cellular stress and limit tissue damage; however, it can also lead to glial scarring.^39^ Tau protein binds microtubules together by stabilising their structures in the neurono-axonal processes; it is mainly located in the brain’s white matter.^40^ Ischemia leads to the hyperphosphorylation of tau, subsequently detaching tau from the microtubules. This detachment creates insoluble accumulations of tau, interrupting axonal transport and causing dysfunction of neuronal signalling.41., 42.

In several small studies, elevated concentrations of GFAp were associated with poor neurological outcomes after CA, but the prognostic ability of GFAp remained poor.8., 9., 43. Ebner et al. determined the prognostic ability of GFAp after CA in a large cohort of 717 patients from the Targeted Temperature Management (TTM) trial.44., 45. The predictive ability of GFAp at 48 h after the arrest appeared good (AUROC 0.88), but at high specificities (>95%), GFAp showed only moderate sensitivity (50% or less). Additionally, GFAp appeared to be more accurate in predicting the neurological outcome compared with NSE at 24, 48, and 72 h after CA. The present study showed similar predictive accuracy for GFAp (AUROC of 0.91 and a sensitivity of 39%), but GFAp was not superior to NSE and did not increase the predictive accuracy when combined with clinical factors and NSE. In the TTM cohort, the overall median GFAp concentrations were lower than those in the present study with a cutoff at 48 h of 142 pg/ml (95% specificity), a difference that may be explained by the disparate analysis methods employed and the distinct study populations included.

Thus far, there is only one large study that focused on tau after CA; the other two are small pilot studies.18., 21., 46. Mattson et al. studied tau in the TTM trial cohort and found that tau had a higher accuracy in predicting neurological outcome compared with NSE at 48 and 72 h after the arrest, with the best accuracy recorded at 72 h.^21^ In their study, the cutoff value obtaining 95% specificity at 72 h was 7.9 ng/l with a good sensitivity of 71%, which is in line with our corresponding cutoff (7.75 pg/ml, sensitivity 88%). In our study the AUROC values of tau were greater than the AUROC values of NSE at 48 and 72 h (Table S4), but the differences were not statistically significant. However, the prognostic model with clinical factors and NSE significantly improved after the addition of tau at 48 and 72 h (Table 3). With an apparent half-life of about 10 h, the kinetics of tau is different from that of NSE with a half-life of 24–48 h.21., 39. We noticed an initial peak (at 0 h) in median tau concentrations in both outcomes but in later samples the concentrations diverged substantially. The initial peak could be a result of the ischemic phase during the arrest, while the later increase in tau concentrations in the unfavourable outcome group could be attributed to secondary damage mechanisms.^41^ A similar two-phased tau release has been noted previously.18., 46. Most likely, the initial release of tau does not indicate poor prognosis. Considering tau kinetics, it could be more useful with serial measurements than at single timepoints.

When determining the feasibility of biomarkers as prognostic tools, high specificity for poor outcome is the priority consideration. However, for a relevant marker, moderate sensitivity is also required. Tau retained good sensitivity with high specificities, and the low tau concentration beyond 48 h after the arrest suggested a favourable outcome. Prognostic accuracy appeared good individually and improved when both markers were used in combination. Thus, we consider GFAp and tau as promising markers in outcome prediction after CA. However, we recently studied neurofilament light (NfL) in the same cohort and found it even more promising after CA with an AUROC of 0.98 at 24, 48, and 72 h.^47^ The high performance of NfL was also noted in the TTM trial cohort.48., 49. Currently, a clinically validated highly sensitive method exists for Nfl in a handful of countries, whereas such is not available for GFAp and tau.^50^ For GFAp, however, the point-of-care testing has been approved for research purposes (through Abbott i-STAT).^51^

Our study has several strengths. The trial was conducted in multiple centres and the patients were treated according to the current guidelines, including targeted temperature management either with 33 or 36 °C. We used an ultrasensitive method in the analysis of the biomarkers.^25^ Our cohort of OHCA patients is rather homogenous, which limits the bias caused by differences in baseline factors but also complicates the generalisation of the results. The major limitation of our study is the small sample size, which exposes our results to type II error. Additionally, the blood samples were collected during the trial, but the trial was not primarily designed for the analysis of the studied markers, which could have reflected the timing of the sampling. The frozen plasma samples were stored up to 3.5 years before the biomarker analysis. Plasma GFAp and tau are shown to be stable after repeated freeze–thaw cycles, which is likely correlated with long-term storage stability.^50^ In a study by Chiu et al. the plasma samples of tau were found to be moderately degraded after 5.4 years of storage at −80 °C, but the degradation did not affect the utility of tau to differentiate patients with cognitive impairment disorders from healthy controls.^52^ We used a different method for tau analysis from that was used in the study by Chiu et al. Furthermore, we found no difference in the prognostic ability of tau between the patients recruited during the first half of the trial and with those recruited during the second half of the trial (results not shown). Thus, we believe that the stability of tau is not an issue in our study.

## Conclusions

Both GFAp and tau performed well in predicting the neurological outcomes after OHCA, but whether they are superior to NSE cannot be concluded based on our study. The combination of both GFAp and tau yielded increased prognostic accuracy compared with either of them alone.

## CRediT authorship contribution statement

**Humaloja Jaana:** Methodology, Validation, Formal analysis, Data curation, Writing – original draft, Writing – review & editing, Visualization. **Lähde Marika:** Investigation, Writing – review & editing. **J. Ashton Nicholas:** Methodology, Formal analysis, Investigation. **Reinikainen Matti:** Conceptualization, Methodology, Resources, Writing – original draft, Supervision. **Hästbacka Johanna:** Conceptualization, Methodology, Writing – review & editing. **Jakkula Pekka:** Conceptualization, Methodology, Investigation, Data curation. **Friberg Hans:** Methodology, Writing – review & editing. **Cronberg Tobias:** Methodology, Writing – review & editing. **Pettilä Ville:** Conceptualization, Methodology, Resources. **Blennow Kaj:** Methodology, Writing – review & editing. **Zetterberg Henrik:** Conceptualization, Methodology, Writing – review & editing. **B. Skrifvars Markus:** Conceptualization, Methodology, Resources, Writing – original draft, Writing – review & editing, Supervision.

## Declaration of Competing Interest

J. Humaloja, M.L., N.J.A, M.R., J. Hästbacka, P.J., H.F., T.C. and V.P. have nothing to disclose. M.B.S. has received a lecture fee and a travel grant from BARD Medical (Ireland and South Korea, not related to this study). H.Z. has served at scientific advisory boards and/or as a consultant for AbbVie, Alector, Eisai, Denali, Roche Diagnostics, Wave, Samumed, Siemens Healthineers, Pinteon Therapeutics, Nervgen, AZTherapies, CogRx and Red Abbey Labs; has given lectures in symposia sponsored by Cellectricon, Fujirebio, Alzecure and Biogen; and is a co-founder of Brain Biomarker Solutions in Gothenburg AB (BBS), which is a part of the GU Ventures Incubator Program (outside submitted work). KB has served as a consultant at advisory boards or at data-monitoring committees for Abcam, Axon, Biogen JOMDD/Shimadzu, Julius Clinical, Lilly, MagQu, Novartis, Prothena, Roche Diagnostics and Siemens Healthineers and is a co-founder of Brain Biomarker Solutions in Gothenburg AB (BBS), which is a part of the GU Ventures Incubator Program, all unrelated to the work presented in this paper.
