# Supplementary material for: GFAp and tau protein as predictors of neurological outcome after out-of-hospital cardiac arrest: A post hoc analysis of the COMACARE trial
Source: Resuscitation. 2022 Jan;170:141–9. doi: 10.1016/j.resuscitation.2021.11.033 (PMC8786666; doi:10.1016/j.resuscitation.2021.11.033)
Supplement: Supplementary data 1 [file mmc1.pdf]

SUPPLEMENTAL MATERIAL

GFAp and tau protein as predictors of neurological outcome after out-of-hospital cardiac arrest: a post hoc analysis of COMACARE trial

Humaloja Jaana, Lähde Marika, Ashton N, Reinikainen Matti, Hästbacka Johanna, Jakkula Pekka, Friberg H6, Cronberg T, Pettilä Ville, Blennow K, Zetterberg Hans, Skrifvars Markus B

Contents

Table S1
Table S2
Table S3
Table S4
Fig. S1
Table S5
Table S6
Table S7
Fig. S2
Table S8

Table S1 Significance of the difference in GFAp and tau concentrations over time between patients with favorable (CPC 1-2) vs unfavorable (CPC 3-5) outcome at 6 months and between the treatment target groups

	Concentration over time	Concentration between groups	Time * Group[a]
**GFAp**			
Outcome (CPC 1-2 vs CPC 3-5)	<0.001	<0.001	<0.001
PaO_2 (10-15 vs 20-25 kPa)	<0.001	1.00	1.00
$PaCO_2$ (4.5-4.7 vs 5.8-6.0 kPa)	<0.001	0.58	0.74
MAP (65-75 vs 80-100 mmHg)	<0.001	0.99	0.85
**Tau**			
Outcome (CPC 1-2 vs CPC 3-5)	<0.001	<0.001	<0.001
PaO_2 (10-15 vs 20-25 kPa)	<0.001	0.57	0.55
$PaCO_2$ (4.5-4.7 vs 5.8-6.0 kPa)	<0.001	0.12	0.16
MAP (65-75 vs 80-100 mmHg)	<0.001	0.40	0.51

[a] Interaction term of time and outcome/treatment target group
Definitions of abbreviations GFAp glial fibrillary acidic protein
CPC cerebral performance category
MAP mean arterial pressure

Table S2 GFAp concentrations for patients between treatment target groups

GFAP concentration pg/ml /median (IQR)

Time	PaO_2 low	PaO_2 high	p-value*	missing
	57	55		
admission	110.6 (63.0-207.8)	151.1 (95.1-260.0)	0.08	0
24 h	236.3 (125.6-922.9)	373.4 (197.6-899.2)	0.22	2
48 h	273.0 (188.1-1189.7)	485.3 (213.0-1458.6)	0.29	4
72 h	294.2 (133.3-918.5)	415.4 (137.4-1243.1)	0.41	8

GFAP concentration pg/ml /median (IQR)

Time	$PaCO_2$ low	$PaCO_2$ high	p-value*	missing
	56	56		
admission	114.8 (69.4-189)	137.6 (82.2-277.5)	0.22	0
24 h	325.8 (141.5-604.4)	305.6 (125.6-1123.5)	0.65	2
48 h	350 (193.7-1183.1)	496.1 (184.1-1290.7)	0.70	4
72 h	300.6 (133.8-922.2)	403 (133.6-1319.4)	0.40	8

GFAP concentration pg/ml /median (IQR)

Time	MAP low	MAP high	p-value*	missing
	56	56		
admission	142.9 (76.4-229.2)	118.1 (71.3-211.6)	0.33	0
24 h	362.2 (197.6-992.3)	233.2 (109.8-894.6)	0.15	2
48 h	441.7 (239.7-1403.5)	268.1 (136.1-1197.5)	0.16	4
72 h	374.5 (210.9-1457.5)	250.6 (129.8-910.5)	0.08	8

* Mann Whitney U

Table S3 Tau concentrations for patients between treatment target groups

Tau concentration pg/ml /median (IQR)

Time	PaO_2 low	PaO_2 high	p-value*	missing
	57	55		
admission	11.0 (6.6-17.9)	9.4 (6.9-27.0)	0.73	0
24 h	5.4 (3.3-12.1)	5.8 (3.3-16.0)	0.47	1
48 h	4.2 (2.7-12.4)	4.3 (2.6-55.1)	0.63	3
72 h	3.7 (2.4-10.3)	5.2 (2.6-78.7)	0.17	7

Tau concentration pg/ml /median (IQR)

Time	$PaCO_2$ low	$PaCO_2$ high	p-value*	missing
	56	56		
admission	11.6 (7.2-18)	9.4 (6-22.4)	0.49	0
24 h	5.2 (3.3-13.7)	6.1 (2.9-15.5)	0.62	1
48 h	3.6 (2.6-9.8)	5.7 (2.6-66.3)	0.11	3
72 h	3.8 (2.2-7.1)	5.4 (2.9-79.3)	0.03	7

Tau concentration pg/ml /median (IQR)

Time	MAP low	MAP high	p-value*	missing
	56	56		
admission	11.3 (7.6-26.6)	10.5 (6.4-17.4)	0.36	0
24 h	6 (3.6-15.9)	5.6 (2.8-12.5)	0.29	1
48 h	4.9 (2.8-35.3)	3.5 (2.5-9.8)	0.14	3
72 h	4.9 (2.9-120.1)	3.9 (2.4-7.3)	0.10	7

* Mann Whitney U

Table S4 Comparison of the abilities of GFAp, tau, and NSE to predict the unfavorable neurological outcome (CPC 3-5) at 6 months.

	AUROC (95 % CI)	AUROC (95 % CI)	*p* value
	GFAp	NSE	
0 h	0.66 (0.55 - 0.76)	0.56 (0.44 - 0.68)	0.28
24 h	0.87 (0.80 - 0.94)	0.77 (0.68 - 0.86)	0.08
48 h	0.91 (0.85 - 0.97)	0.86 (0.79 - 0.94)	0.29
72 h	0.91 (0.85 - 0.96)	0.90 (0.82 - 0.97)	0.78
	GFAp	tau	
0 h	0.66 (0.55 - 0.76)	0.59 (0.48 - 0.70)	0.37
24 h	0.87 (0.80 - 0.94)	0.82 (0.73 - 0.91)	0.32
48 h	0.91 (0.85 - 0.97)	0.93 (0.86 - 0.99)	0.74
72 h	0.91 (0.85 - 0.96)	0.95 (0.89 - 1.00)	0.33
	tau	NSE	
0 h	0.59 (0.48 - 0.70)	0.56 (0.44 - 0.68)	0.68
24 h	0.82 (0.73 - 0.91)	0.77 (0.68 - 0.86)	0.28
48 h	0.93 (0.86 - 0.99)	0.86 (0.79 - 0.94)	0.16
72 h	0.95 (0.89 - 1.00)	0.90 (0.82 - 0.97)	0.17

Definitions of abbreviations GFAp glial fibrillary acidic protein
AUROC area under the receiver operating characteristic curve
95 % CI 95 % confidence interval
NSE neuron specific enolase
CPC cerebral performance category

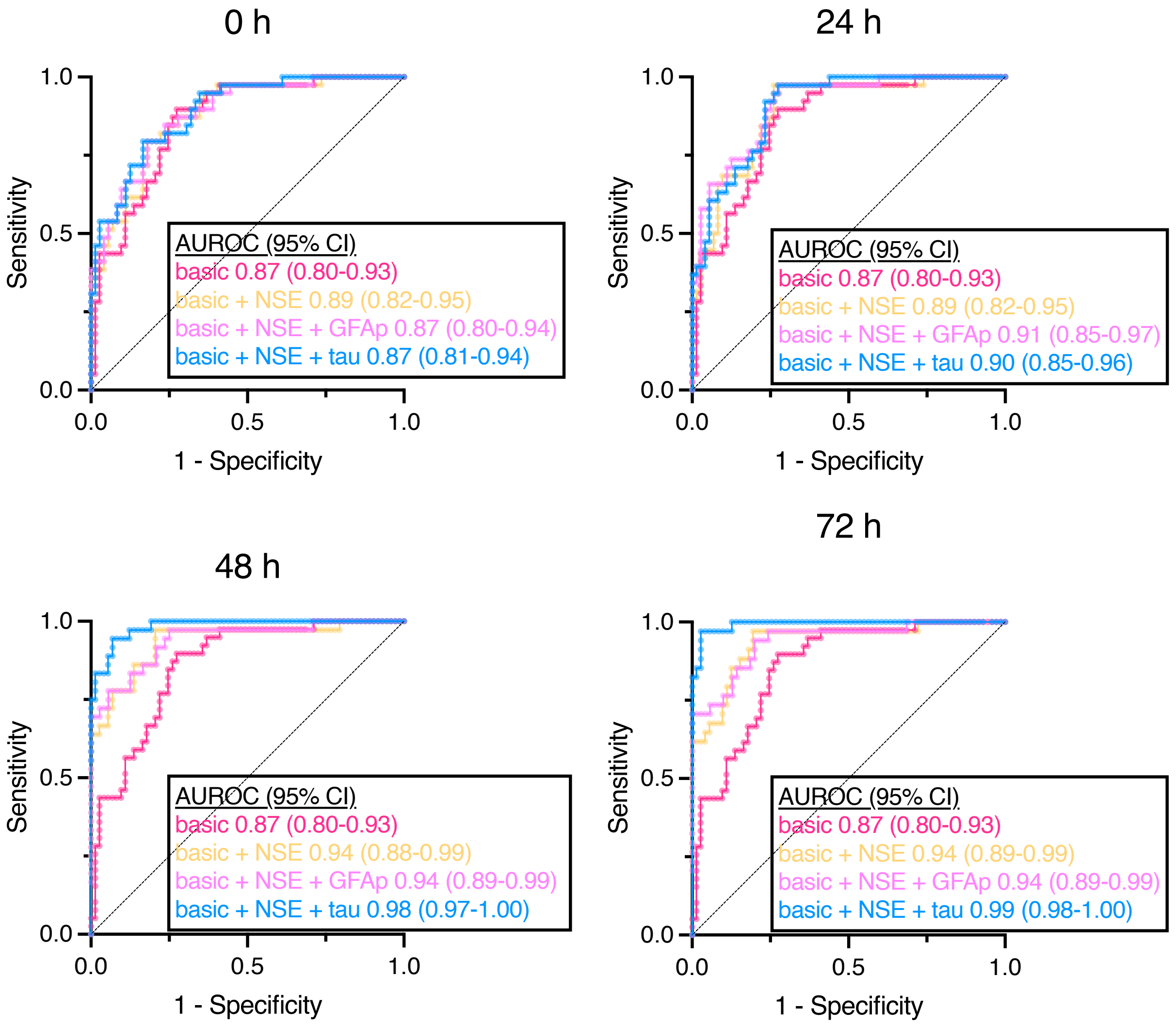

Fig S1. Receiver operating characteristic curves and areas under the curves (AUROC) with 95% confidence intervals (CI) for multiple logistic regression models with clinical signs and NSE, GFAp and tau at different time points predicting unfavorable neurological outcome at six months.

Basic model includes patient age, delay to the return of spontaneous circulation, and receipt of bystander-given life support. All other models are created adding the biomarker concentration at different time-points into the model. Definitions of abbreviations: ROC receiving operating characteristic, AUROC area under the receiving operating characteristic curve, CI confidence interval GFAp glial fibrillary acidic protein, Tau protein tau, CPC cerebral performance category

Table S5 Logistic regression model to predict unfavorable neurological outcome (CPC 3 – 5) at 6 months

**Baseline model**	**OR for unfavorable outcome**	**CI 95%**	*p* value
Age (years)	1.06	1.01–1.11	0.016
ROSC (minutes)	1.20	1.11–1.30	<0.001
Bystander resuscitation (yes)	0.20	0.05–0.73	0.015
**Baseline model + NSE**			
NSE 0h	1.02	0.98–1.05	0.30
NSE 24 h	1.06	1.02–1.11	0.005
NSE 48 h	1.10	1.04–1.17	0.001
NSE 72 h	1.11	1.03–1.19	0.004
**Baseline model + NSE + GFAp**			
GFAp 0 h	1.00	1.00-1.00	0.12
GFAp 24 h	1.00	1.00-1.00	0.008
GFAp 48 h	1.00	1.00-1.01	0.064
GFAp 72 h	1.01	1.00-1.00	0.056
**Baseline model + NSE + tau**			
Tau 0 h	0.99	0.98–1.00	0.08
Tau 24 h	1.04	0.99–1.08	0.12
Tau 48 h	1.28	1.06–1.53	0.01
Tau 72 h	1.43	1.08–1.89	0.014

Table S6 Cutoff values to separate unfavorable and favorable neurological outcome based on Youden index and 95%, 97%, and 99% specificity with corresponding sensitivities, positive predictive value, negative predictive value, and positive likelihood ratio.

GFAp	cutoff [pg/ml]	specificity (95 % CI)	sensitivity (95 % CI)	PPV (95 % CI)	NPV (95 % CI)	LR+ (95 % CI)	*P*
0 h							
Youden	118	0.53 (0.42-0.65)	0.77 (0.64-0.90)	0.47 (0.35-0.59)	0.81 (0.70-0.92)	1.65 (1.22-2.23)	0.003
95 %	354	0.95	0.26 (0.12-0.39)	0.71 (0.48-0.95)	0.70 (0.61-0.79)	4.68 (1.57-13.95)	0.005
97 %	707	0.97	0.13 (0.02-0.23)	0.71 (0.38-1.05)	0.68 (0.59-0.77)	4.68 (0.95-23.0)	0.049
99 %	3330	0.99	0	0	0.65 (0.56-0.74)	0	1.00
24 h							
Youden	459	0.85 (0.76-0.93)	0.82 (0.69-0.94)	0.74 (0.61-0.87)	0.90 (0.82-0.97)	5.34 (3.04-9.39)	<0.001
95 %	1307	0.95	0.44 (0.29-0.61)	0.81 (0.64-0.98)	0.76 (0.68-0.85)	8.05 (2.92-22.24)	<0.001
97 %	3804	0.97	0.26 (0.12-0.40)	0.97 (0.93-1.01)	0.71 (0.62-0.80)	9.47 (2.19-41.1)	<0.001
99 %	8018	0.99	0.13 (0.02-0.24)	0.83 (0.54-1.13)	0.68 (0.59-0.77)	9.47 (1.15-78.2)	0.02
48 h							
Youden	761	0.90 (0.83-0.97)	0.86 (0.75-0.97))	0.82 (0.69-0.94)	0.93 (0.87-0.99)	8.86 (4.33-18.12)	<0.001
95 %	1798	0.95	0.39 (0.23-0.55)	0.82 (0.64-1.00)	0.76 (0.67-0.85)	9.33 (2.86-30.4)	<0.001
97 %	2858	0.97	0.31 (0.16-0.46)	0.85 (0.65-1.04)	0.74 (0.65-0.83)	11 (2.57-47.0)	<0.001
99 %	6262	0.99	0.19 (0.07-0.32)	0.88 (0.65-1.10)	0.71 (0.62-0.80)	14 (1.79-109.5)	0.002
72 h							
Youden	355	0.71 (0.61-0.82)	0.97 (0.91-1.00)	0.62 (0.49-0.75)	0.98 (0.94-1.02)	3.40 (2.3-4.94)	<0.001
95 %	1394	0.95	0.53 (0.36-0.70)	0.86 (0.71-1.01)	0.81 (0.72-0.89)	12.35 (3.9-39.1)	<0.001
97 %	1500	0.97	0.50 (0.33-0.67)	0.89 (0.76-1.03)	0.80 (0.71-0.89)	0.51 (0.37-0.72)	<0.001
99 %	4235	0.99	0.29 (0.14-0.45)	0.91 (0.74-1.08)	0.74 (0.65-0.83)	20.59 (2.8-154.3)	<0.001

Tau	cutoff [pg/ml]	specificity (95 % CI)	sensitivity (95 % CI)	PPV (95 % CI)	NPV (95 % CI)	LR+ (95 % CI)	*P*
0 h							
Youden	7.98	0.40 (0.29-0.51)	0.79 (0.67-0.92)	0.41 (0.30-0.52)	0.78 (0.65-0.92)	1.32 (2.03-1.69)	0.06
95 %	88.79	0.95	0.08 (0.00-0.16)	0.43 (0.06-0.80)	0.66 (0.57-0.75)	1.40 (0.33-5.96)	0.69
97 %	138	0.97	0.08 (0.00-0.16)	0.6 (0.17-1.03)	0.66 (0.57-0.75)	2.81 (0.49-16.1)	0.34
99 %	206	0.99	0	0	0.65 (0.56-0.74)	0	1.00
24 h							
Youden	10.30	0.86 (0.78-0.94)	0.68 (0.54-0.83)	0.72 (0.58-0.87)	0.84 (0.76-0.92)	4.99 (2.70-9.24)	<0.001
95 %	15.80	0.95	0.47 (0.31-0.63)	0.82 (0.66-0.98)	0.78 (0.69-0.86)	8.64 (3.15-23.7)	<0.001

97 %	37	0.97	0.26 (0.12-0.40)	0.83 (0.62-1.04)	0.71 (0.63-0.81)	9.61 (2.22-41.6)	<0.001
99 %	40	0.99	0.21 (0.08-0.34)	0.89 (0.68-1.09)	0.71 (0.62-0.79)	15.37 (2.00-118.4)	<0.001
48 h							
Youden	8.03	0.92 (0.85-0.98)	0.86 (0.75-0.97)	0.84 (0.72-0.96)	0.93 (0.87-0.99)	10.48 (4.81-22.8)	<0.001
95 %	9.86	0.95	0.81 (0.68-0.93)	0.88 (0.77-0.99)	0.91 (0.84-0.97)	14.70 (5.59-38.6)	<0.001
97 %	15.0	0.97	0.75 (0.61-0.89)	0.93 (0.84-1.02)	0.89 (0.82-0.96)	27.38 (6.89-108.8)	<0.001
99 %	16.0	0.99	0.75 (0.61-0.89)	0.93 (0.84-1.02)	0.89 (0.82-0.96)	27.38 (6.89-108.8)	<0.001
72 h							
Youden	10.56	1.00 (1.00-1.00)	0.88 (0.77-0.99)	1.00 (1.00-1.00)	0.95 (0.90-1.00)	infinity	<0.001
95 %	7.75	0.95	0.88 (0.77-0.99)	0.88 (0.77-0.99)	0.94 (0.89-1.00)	15.66 (6.00-40.9)	<0.001
97 %	9.0	0.97	0.88 (0.77-0.99)	091 (0.81-1.01)	0.94 (0.89-1.00)	20.9 (6.85-63.6)	<0.001
99 %	10	0.99	0.88 (0.77-0.99)	1	0.95 (0.90-1.00)	infinity	<0.001

Definitions of abbreviations

GFAp glial fibrillary acidic protein

AUROC area under the receiver operating characteristic curve

95 % CI 95 % confidence interval

PPV positive predictive value

NPV negative predictive value

LR+ positive likelihood ratio

Table S7 Cutoff values for GFAp and tau at 48 and 72 h after cardiac arrest to predict favorable neurological outcome set at high sensitivities with corresponding negative predictive values and specificities.

GFAp	Cut off [pg/ml]	sensitivity (95 % CI)	NPV (95 % CI)	specificity (95 % CI)	p-value
48 h					
100 % sensitivity	210	1.00 (1.00-1.00)	1.00 (1.00-1.00)	0.43 (0.32-0.54)	<0.001
95 % sensitivity	439	0.94 (0.87-1.00)	0.96 (0.92-1.00)	0.75 (0.65-0.85)	<0.001
72 h					
100 % sensitivity	187	1.00 (1.00-1.00)	1.00 (1.00-1.00)	0.44 (0.33-0.56)	<0.001
95 % senstivity	359	0.94 (0.86-1.00)	0.96 (0.91-1.00)	0.73 (0.62-0.83)	<0.001

Tau	Cut off [pg/ml]	se sensitivity (95 % CI)	NPV (95 % CI)	specificity (95 % CI)	p-value
48 h					
100 % sensitivity	*NA*	*patient with poor outcome had the smallest tau concentration at 48 h*			
95 % sensitivity	3.28	0.94 (0.87-1.00)	0.95 (0.89-1.00)	0.53 (0.42-0.65)	<0.001
72 h					
100% sensitivity	2.10	1.00 (1.00-1.00)	1.00 (1.00-1.00)	0.21 (0.12-0.31)	0.002
95 % sensitivity	3.37	0.94 (0.86-1.00)	0.95 (0.88-1.00)	0.52 (0.40-0.64)	<0.001

Definitions of abbreviations GFAp glial fibrillary acidic protein
95 % CI 95 % confidence interval
NPV negative predictive value

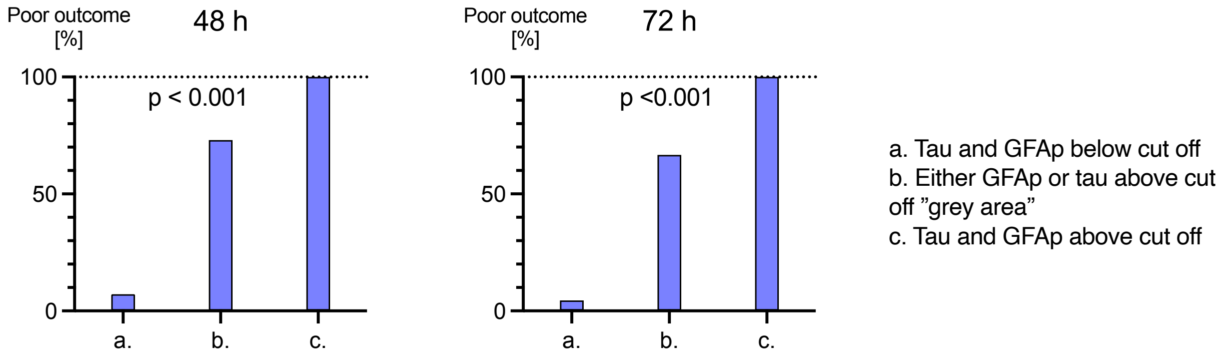

Fig S2. Proportion of patients categorised correctly as having unfavourable outcomes at six months in scenarios a., b., and c.
Poor outcome was determined as Cerebral Performance Category 3 – 5 at 6-months. Cutoffs with 95% specificity were used and they were 1798 pg/ml and 1394 pg/ml for GFAp at 48 and 72h respectively, and 9.86 pg/ml and 7.75 pg/ml for tau respectively. P-values are computed with Pearson’s Chi Squared test.

Table S8 Proportion of patients correctly predicted as having unfavourable outcomes from those who exceeded the 48 and 72 h cutoff for poor prognosis for GFAp and tau in different scenarios

	Markers above the cutoff			
	GFAp only	Tau only	GFAp or tau[B]	GFAp and tau[C]
Cutoff 48h (pg/ml)*	1798	9.86		
No. Of patients above the 48h cutoff	5	21	26	12
Patients above the cutoff with poor 6-month outcome**	2 (40%)	17 (81%)	19 (73%)	12 (100%)
Cutoff 72h (pg/ml)*	1394	7.75		
No. Of patients above the 72h cutoff	4	17	21	17
Patients above the cutoff with poor 6-month outcome*	1 (25%)	13 (76%)	14 (67%)	17 (100%)

[B] corresponds to a scenario B in Fig. S2
[C] corresponds to a scenario C in Fig. S2
* Cutoff with specificity of 95%
**Fraction of total refers to percentage of patients correctly predicted as having poor outcomes of those who exceeded the cutoff
